# Sleeve Gastrectomy Provides Cardioprotection from Oxidative Stress In Vitro Due to Reduction of Circulating Myeloperoxidase

**DOI:** 10.3390/nu15224776

**Published:** 2023-11-14

**Authors:** Matthew Barron, Hailey Hayes, Zachary Bice, Kirkwood Pritchard, Tammy Lyn Kindel

**Affiliations:** 1 Department of Surgery, Division of Gastrointestinal and Minimally Invasive Surgery, Medical College of Wisconsin, 8900 W. Doyne Avenue, Milwaukee, WI 53226, USA; mbarron@mcw.edu (M.B.); haihayes@iu.edu (H.H.); 2 Division of Pediatric Surgery, Children’s Research Institute, Milwaukee, WI 53226, USA; zbice@mcw.edu (Z.B.); kpritch@mcw.edu (K.P.)

**Keywords:** heart failure, myeloperoxidase, bariatric surgery, sleeve gastrectomy, oxidative stress

## Abstract

Bariatric surgery, including sleeve gastrectomy (SG), improves systolic and diastolic function, which is independent of weight loss in rodent models. The cause of weight loss-independent improvements in cardiac function are unknown but may originate from the gastrointestinal tract. In this study, we investigated whether a circulating blood factor is a mechanism for acute cardioprotection after SG by testing the utility of rodent SG plasma to reduce metabolic stress in vitro. For the initial experiment, obese male Zucker rats underwent SG, ad lib sham, or pair-fed sham surgeries (n = six SG, n = eight SH, n = eight PF). For all other studies, a second group of Zucker rats underwent SG or ad lib sham surgeries (n = eight SH, n = six SG). Six weeks following surgery, plasma was collected from each group, both in the fasting and post-prandial (pp) state. This plasma was then pooled per surgical group and nutrient state and tested in multiple in vitro cell culture and extra-cellular assays to determine the effect of SG on myotubular metabolic stress compared to the sham surgeries. Post-prandial SG plasma (ppSG), but not fasting SG, pp, or fasting sham plasma, reduced the metabolic stress of the H9c2 cells as measured by lactate dehydrogenase (LDH) release (*p* < 0.01). Unlike SG, weight reduction through pair-feeding did not prevent H9c2 metabolic stress. The PpSG plasma had the slowest rate of extracellular hydrogen peroxide consumption and peroxidatic activity compared to the pp sham, fasting SG, and fasting sham groups. Redox testing of plasma with aminiobenzoic acid hydrazide and edaravone suggested a pattern supporting myeloperoxidase (MPO), or other peroxidases, as the primary component responsible for reduced metabolic stress with ppSG plasma. The PpSG plasma contained 35% less circulating MPO protein as compared to the pp sham and fasting SG plasma. The plasma from an MPO global knockout rat also prevented metabolic stress of the H9c2 cells, compared to the significant increase in LDH release from the plasma of the WT controls (*p* < 0.01). The MPO global knockout plasma also had a rate of extracellular hydrogen peroxide consumption and peroxidatic activity comparable to the ppSG plasma. These studies suggest that one of the weight loss-independent mechanisms by which SG improves myocellular function could be a reduced pro-oxidative environment due to lower circulating levels of MPO. It appears that the gastrointestinal tract is of critical importance to these findings, as the MPO levels were only lowered after enteral, nutrient stimulation in the SG rats. If this surgical effect is confirmed in humans, SG may be a unique surgical treatment for multiple diseases with a pathogenesis of inflammation and oxidative damage, including obesity-associated heart failure with preserved ejection fraction.

## 1. Introduction

Obesity is a disease of excess nutrient intake and storage which creates systemic low-grade inflammation [1]. Studies have shown the occurrence of oxidative stress, resulting from increased production and/or diminished metabolism of reactive oxygen species (ROS) in obese patients [2,3], as well as obesity-associated conditions like type 2 diabetes mellitus, atherosclerosis, and obesity-associated heart failure with preserved ejection fraction (HFpEF). HFpEF currently affects more than 3 million people in the US and is projected to reach 8 million (3% of the population 18 years and older) by the year 2030 [4]. HFpEF is commonly associated with pulmonary disease, anemia, frailty, hypertension, and atrial fibrillation [4]. Pro-inflammatory obesity-associated medical conditions may promote HFpEF through cardiac microvascular endothelial cell inflammation and impaired processes that clear dysfunctional proteins (unfolded protein response). This results in myocardial structural and functional abnormalities with coronary microvascular dysfunction [5]. In addition, increased oxidative stress with decreased nitric oxide–soluble guanylate cyclase-protein kinase G signaling may also play a role [6]. It seems clear that multiple biological pathways result in HFpEF, requiring distinct therapies.

Sleeve gastrectomy (SG), the most performed bariatric surgery in the United States, almost universally improves diastolic dysfunction, a common finding in HFpEF [7]. Clinical studies have shown that in addition to improvements in diastolic function, SG ameliorates the symptoms of HFpEF, reverses adverse LV remodeling, and is associated with alterations in the plasma lipidome [8]. We have previously found that diastolic dysfunction after SG improves independent of weight loss or calorie restriction in an obese animal model suggesting a mechanism originating from the gastrointestinal tract [9].

Several gastrointestinal-mediated changes occur after SG which could all be potential mechanisms for improvement in HFpEF after surgery. Alterations to the gut microbiome itself are increasingly recognized as an important mechanism in the benefits of SG, which are likely independent of caloric restriction [10]. SG also removes ghrelin-producing cells in the stomach resulting in decreased circulating ghrelin, as well as increased secretion of intestinal glucagon-like peptide-1 [11]. SG increases systemic bile acid levels and alters bile acid composition [12], which signals through nuclear receptors such as the farnesoid X receptor to facilitate metabolic benefits.

Given multiple weight loss-independent mechanisms exist from the gastrointestinal tract after SG, we hypothesized that, rather than weight loss, a systemic circulating factor originating from the gastrointestinal tract may be responsible for the improvement in cardiac function found in our prior in vivo animal studies [13,14,15]. If this hypothesis is true, this circulating factor could be harnessed as a unique pharmacologic treatment of HFpEF or support gastrectomy as a novel surgical therapy for HFpEF independent of weight loss effects. The aim of our present study was to investigate whether a circulating blood factor post-SG could acutely provide cellular protection of cardiomyocytes from oxidative stress in vitro.

## 2. Materials and Methods

### 2.1. Surgical Study Designs

This study and all procedures were approved by the Medical College of Wisconsin’s Institutional Animal Care and Use Committee (Animal Use Agreement #00005343). Portions of this study involve blood samples from rats that had undergone SG to assess the relationship between hypertension and gut microbial changes as previously published [7]. As a review, obese (strain 185) male Zucker rats were purchased at 5 weeks of age (Charles River Laboratories, Wilmington, MA, USA). All rats were housed in pairs prior to surgery and started on Purina Diet 5008 at 8 weeks of age. In the first cohort, Zucker rats were stratified into three different surgical groups: obese SG, obese pair-fed sham, and obese ad lib sham [7]. In a second separate cohort, Zucker rats were stratified into two surgical groups: obese SG and obese ad lib sham, matched for body weight. After surgery, the animals were individually caged and resumed Purina diet 5008 72 h after surgery until the study completion. Animals were euthanized with collection of blood at 6 weeks post-surgery in the first cohort [7]. For the second cohort, animals were fasted overnight. The following day, half of each surgical group were given a 10% glucose gavage, creating post-prandial and fasting sub-groups. Blood was collected 15 min following gavage. Blood was collected from the inferior vena cava in EDTA tubes and spun at 1000 g for 15 min to separate plasma. Plasma was transferred to fresh tubes, pooled as groups or sub-groups, and stored @ −80 °C until experimentation. 

### 2.2. Construction of Myeloperoxidase (MPO) Knockout (KO) Rat Model

A novel strain of SD rats was created through the injection of a SpCas9/sgRNA CRISPR ribonucleoprotein targeting AGGGCCACGTGCAGATAGTCGG within the MPO gene of Crl:SD (Charles River Laboratories) rat embryos via pronuclear injection. The founder animals were screened using fluorescent fragment analysis of the targeted locus (primers: 5′GAGTTCTTTCTGCCTGCTCACCG3′ and 5′GAGTTCTTTCTGCCTGCTCACCG3′). The genotyping results were further verified by Sanger sequencing to confirm the accuracy of the introduced mutations. One founder harboring an 11-base pair deletion (rn7: chr10:72595923-72595933) within exon 4 was back crossed with the parental strain to establish a stable genetic line. Subsequent litters were screened using fluorescent fragment analysis to ensure the presence of the introduced mutations. This strain was registered as SD-Mpo^em1Mcwi^ (RGDID: 401717572).

### 2.3. In Vitro Oxidative Stress Culture Assay

H9c2(2-1) embryonic rat myocardium cells (ATCC) were cultured at a density of 40,000 cells/well in a 24-well plate the night before the experiment in either 1% plasma (from surgical cohorts 1, 2, or MPO KO rats) or fetal bovine serum (FBS) in high-glucose (4.5 g/L) Dulbecco’s Modified Eagle Medium (DMEM, ThermoFisher, Waltham, MA, USA). The morning of the experiment, cells were subjected to oxidative/metabolic stress from a modified protocol previously published, with 200 µM hydrogen peroxide (H_2_O_2_) and 10 mM deoxy-glucose in 160 µL glucose-free DMEM (ThermoFisher) for 1 h at 37 °C, with 5% CO_2_ [16]. Unstressed controls were cultured for this hour in the same volume of 1% plasma or FBS in high-glucose medium without H_2_O_2_. Cells were then allowed to recover for a 3 h period after the addition of 400 µL high-glucose medium containing 1% plasma or FBS. Lactate dehydrogenase (LDH) levels were measured as an indication of oxidative stress damage at several timepoints within the assay, and/or following the recovery period using the Cytotoxicity Detection Kit (LDH; Millipore Sigma, Burlington, MA, USA). All determinations were performed in triplicate.

### 2.4. Redox Measurements Using Fluorescence Plate Read

Plasma from the second cohort of rats was evaluated for its ability to metabolize H_2_O_2_ using coumarin-7-boronic acid (CBA) as a probe for H_2_O_2_, and a plate reader-based monitoring of CBA oxidation [17]. For CBA experiments, a 96-well plate was loaded with 160 µL/well of 0.25 mM CBA in HBSS/25 mM HEPES/0.1 mM dtpa. Next, 20 µL/well of 10× plasma samples in HBSS/25 mM HEPES/0.1 mM dtpa was added, along with 20 µL/well of 1.0 mM H_2_O_2_ in water. Immediately following the addition of H_2_O_2_, fluorescence (excitation wavelength: 355 nm; emission wavelength: 460 nm) was measured for a 2 h period at room temperature, and the ability of plasma samples to inhibit CBA oxidation was determined. To monitor peroxidatic activity in the plasma samples, H_2_O_2_-induced oxidation of Amplex Red to red fluorescent resorufin was used [18]. For these experiments, 160 µL/well of 62.5 µM Amplex Red was used, followed by the addition of plasma samples and H_2_O_2_, as described above for the CBA-based assay. Finally, for Amplex Red experiments with specific peroxidase inhibitors, wells were loaded with 20 µL/well of 10× plasma samples ±1 mM aminiobenzoic acid hydrazide (ABAH), NaN_3_, or edaravone, 160 µL/well of 62.5 µM Amplex Red in HBSS/25 mM HEPES/0.1 mM dtpa, and 20 µL/well of 1.0 mM H_2_O_2_ in water. Fluorescence (excitation wavelength: 535 nm; emission wavelength: 585 nm) was measured for a 2 h period at room temperature as before. All determinations were performed in quadruplicates.

### 2.5. Hydroethidine (HE) Oxidation Assay

Plasma from the second cohort of rats was evaluated for its ability to catalyze H_2_O_2_-induced oxidation of the HE redox probe. HE (40 µM) was incubated for 30 min at room temperature with 100 µM H_2_O_2_ in HBSS/25 mM HEPES/0.1 mM dtpa in the presence or absence of 0.1% plasma samples and immediately injected for HPLC analysis. The products’ profiles were analyzed by HPLC equipped with ultraviolet–vis absorption and fluorescence detectors, as described previously [19].

### 2.6. Western Blot

Western blot analysis was performed on plasma from the second cohort of rats. Briefly, each plasma sample was diluted 1:5 in PBS with protease and phosphatase inhibitors (Millipore Sigma). The Bradford assay was utilized to assess protein concentration in the dilute plasma, and samples were subsequently prepared for gel electrophoresis under reducing conditions. A total of 30 μg of protein from each sample was loaded into a polyacrylamide gel (Bio-Rad, Hercules, CA, USA) and run at 65 V for 10 min, followed by 185 V for 30 min. The gel was photoactivated for stain-free total protein analysis and transferred to a polyvinylidene difluoride membrane (Bio-Rad). After transfer, the membrane was imaged for total protein before being blocked for 15 min at room temperature in blocking buffer (Bio-Rad). The membrane was then probed overnight at 4 °C with MPO primary antibody (SC-33596) at a 1:500 dilution. On the following day, the membrane was probed with anti-rabbit HRP-conjugated secondary antibody (Bio-Rad #1706515) for 1 h at room temperature. Chemiluminescent substrate (Bio-Rad #1705061) was applied to the membrane and the blot was imaged on the Chemidoc MP Imaging System. After image acquisition, band and total protein optical density were determined using Bio-Rad’s Image Lab 6.1 software for Windows.

### 2.7. Statistical Analysis

All values were presented as the mean ± standard deviation. Comparisons of results within surgical groups with different cell culture conditions (stress vs. no-stress, fasting vs. post-prandial), or between two groups only were performed as a student’s *t*-test. A one-way analysis of variance (ANOVA) was performed to determine differences between groups at the same time point and the least significant difference post-hoc analysis for statistically significant F statistics values was determined statistically significant if *p* < 0.05.

## 3. Results

### 3.1. Plasma from Zucker Rats Undergoing SG Is Protective against Oxidative/Metabolic Stress and Weight Loss-Independent

**Figure 1:**
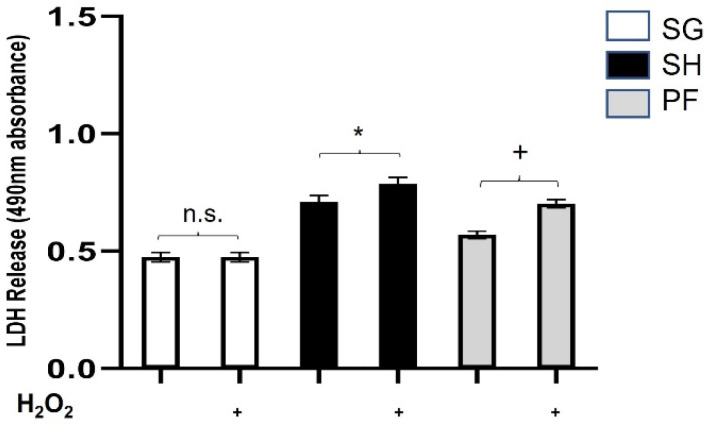
**Plasma from Zucker rats undergoing sleeve gastrectomy (SG) is protective against oxidative/metabolic stress and weight-loss independent.** Lactate dehydrogenase (LDH) secretion was measured from H9c2 cells pre-treated with 1% plasma in DMEM from obese Zucker rats who had undergone SG (SG), ad-lib sham (SH), or pair-fed sham (PF) surgeries. LDH release was measured as 490 nm absorbance under stressed (200 μM H_2_O_2_ and 10 mM deoxy-glucose in DMEM) or unstressed conditions (high glucose DMEM). (*) represents a significant difference in LDH secretion from stressed compared to unstressed within each group (*p* < 0.03). (+) represents a significant difference in LDH secretion from stressed compared to unstressed within each group (*p* < 0.01). Data presented as the mean ± SD, plasma pooled per sub-group (n = 6 SG, n = 8 SH, n = 8 PF) and run in triplicate. n.s. = not statistically significant.

We utilized an in vitro assay to investigate the effects of oxidative/metabolic stress on H9c2 cellular LDH secretion. This assay consistently showed an increase in LDH production when the H9c2 cells were challenged with H_2_O_2_ and low glucose conditions (Supplemental Figure S1). To optimize this assay, we determined a H_2_O_2_ dose and time response (Supplemental Figure S2) from 0 to 500 µM H_2_O_2_. As shown, levels above 200 µM were highly toxic; therefore, 200 µM was chosen for all future experiments. Using these parameters, we tested whether post-prandial plasma from Zucker rats from Cohort 1 could protect the H9c2 cells against oxidative/metabolic damage (using plasma pooled per group and run in triplicate) in a weight loss-independent manner. As previously published, there are significant differences in body weight between obese SG and ad lib, obese sham Zucker groups at 6 weeks, with no difference in the body weights between pair-fed, obese sham and obese SG Zucker groups at 6 weeks [7]. In addition, we have previously published no significant difference in non-fasted cholesterol, free fatty acid, glucose, insulin, or renin concentrations between obese SG, obese ad lib, or obese, pair-fed sham groups to account for the effect in our cell culture model [7]. As shown in Figure 1, the cells exposed to SG plasma had no difference in LDH secretion with stress compared to the unstressed control (minus H_2_O_2_), while the cells exposed to pair-fed sham and ad lib sham plasma expressed higher levels of LDH under stress (*p* < 0.03 for both, respectively). The percent cytotoxicity is shown in Supplemental Figure S3. This suggests that plasma from SG uniquely protects H9c2 cells from oxidative/metabolic stress as measured by LDH in a weight loss-independent manner. 

### 3.2. Protection against Oxidative/Metabolic Stress by SG Plasma Is Acute and Nutrient-Dependent

**Figure 2:**
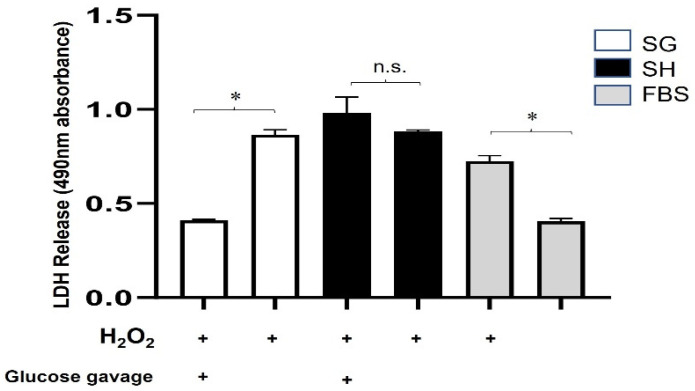
**Protection against oxidative/metabolic stress by sleeve gastrectomy (SG) plasma is acute and nutrient-dependent.** Lactate dehydrogenase (LDH) secretion was measured as 490 nm absorbance from H9c2 cells pre-treated with 1% plasma in DMEM from obese Zucker rats who had undergone SG or Sham (SH) surgery under fasting or 15 min following a 10% glucose gavage with FBS as a control. The sub-groups were tested under stressed (200 μM H_2_O_2_ and 10 mM deoxy-glucose in DMEM) or unstressed conditions (high glucose DMEM). (*) represents a significant difference in LDH secretion from stressed compared to unstressed within each group (*p* < 0.05). Data presented as the mean ± SD, plasma pooled per sub-group (n = 8 SH, n = 6 SG) and run in triplicate. n.s. = not statistically significant.

To determine if SG plasma reduces oxidative/metabolic damage in a nutrient-dependent manner, we repeated the cell culture assay using fasting or post-prandial plasma as described in the methods for Cohort 2. As shown in Figure 2, the LDH levels were significantly lower from the cells exposed to post-prandial SG plasma than from fasting SG plasma under stress conditions (from plasma pooled per sub-group and run in triplicate, *p* < 0.01). The percent cytotoxicity is shown in Supplemental Figure S4. There was no difference in LDH secretion between the post-prandial sham or fasting sham plasma. These data indicate that the beneficial plasma factor on oxidative/metabolic stress is nutrient-dependent. In addition, because the protective effect was observed using plasma collected just 15 min post-gavage, it was an acute effect. 

### 3.3. Plasma-Induced Reduction in Oxidative/Metabolic Cellular Stress after SG Is Not Transient

**Figure 3:**
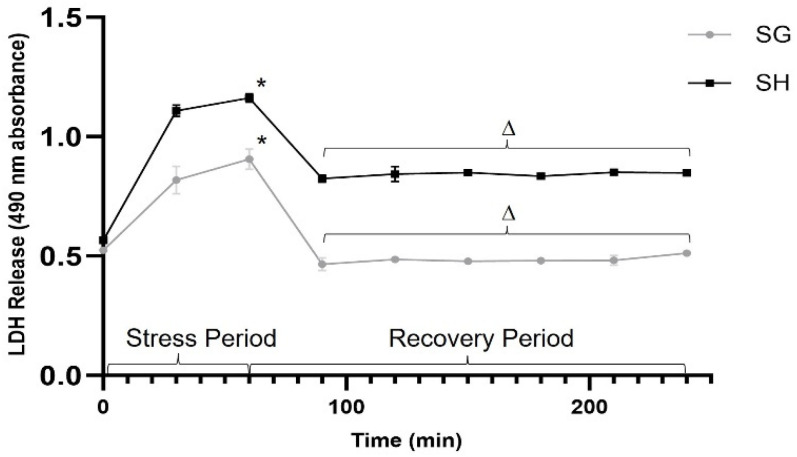
**Plasma-induced reduction in oxidative/metabolic cellular stress after sleeve gastrectomy (SG) is not transient.** 160 μL 1% plasma in stress medium (200 μM H_2_O_2_, 10 mM deoxyglucose in “no glucose” DMEM) was administered at time “0” and cells were incubated for a 60-min stress period. Following this, 400 μL 1% plasma in “high glucose” DMEM was added, and cells were cultured for a 3-h recovery period. Lactate dehydrogenase (LDH) secretion is shown as 490nm absorbance. (*, ∆) represents a significant difference in LDH secretion between groups (*p* < 0.05). Data presented as the mean ± SD, plasma pooled per sub-group (n = 8 SH, n = 6 SG) and run in triplicate.

To further investigate the protective effect of SG plasma, we tested cellular stress over time (plasma pooled per group and run in triplicate). We challenged the H9c2 cells with H_2_O_2_ for a one-hour interval followed by a three-hour recovery period, measuring the LDH levels every 30 min. As shown in Figure 3, an initial rise in LDH activity occurred during the exposure to H_2_O_2_, followed by a subsequent decrease upon the addition of growth medium (to begin the recovery period) in both the samples due to a dilutional effect on LDH. However, the levels of LDH were consistently lower in the presence of post-prandial SG plasma starting at one hour, as compared to the post-prandial sham plasma throughout the assay (*p* < 0.01), indicating that the reduction in H_2_O_2_-mediated stress by SG plasma exposure was non-transient. 

### 3.4. Post-Prandial SG Plasma Has a Slow Rate of Hydrogen Peroxide Consumption

**Figure 4:**
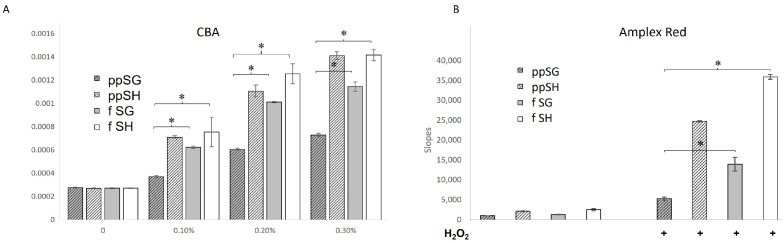
**Post-prandial plasma from sleeve gastrectomy (ppSG) has the slowest rate of hydrogen peroxide oxidation.** (**A**) Oxidation rates of H_2_O_2_ by plasma ranging from 0 to 0.30% using coumarin-7-boronic acid (CBA). ppSG plasma had the slowest rate of hydrogen peroxide oxidation at all plasma levels tested compared to ppSham (ppSH), fasting SG (fSG), and fasting sham (fSH) groups. (**B**) Catalyzation of H_2_O_2_-induced Amplex Red oxidation using plasma ranging from 0 to 0.20% as detected by Amplex Red. As with the CBA probe, ppSG plasma produces the slowest rate among plasma tested, indicating lower damaging oxygen free-radical production. Data presented as the mean ± SD, plasma pooled per sub-group and run in quadruplicate. Statistical significance determined at *p* < 0.05 (*).

To determine if the beneficial effect of post-prandial SG plasma was extracellular or intracellular, we utilized redox probes in an extracellular model. We first tested the ability of plasma to extracellularly consume H_2_O_2_, by testing the effect of plasma on the rate of H_2_O_2_-induced oxidation of CBA, using plasma concentrations from 0 to 0.30%. Based on a simple competition kinetics reasoning, we expected that the consumption of H_2_O_2_ by post-prandial SG plasma would result in faster completion of CBA oxidation, reflected in an increased apparent first-order rate constant. Unexpectedly, as shown in Figure 4A, post-prandial SG plasma resulted in the smallest acceleration of CBA oxidation of any sub-group at all the concentrations tested. This indicates that post-prandial SG plasma consumes H_2_O_2_ at the lowest rate among the plasma samples tested, and the mechanism for reduced cytotoxicity with stress is not direct H_2_O_2_ scavenging by plasma.

### 3.5. Peroxidase Activity Is Uniquely Decreased in Post-Prandial SG Plasma

**Figure 5:**
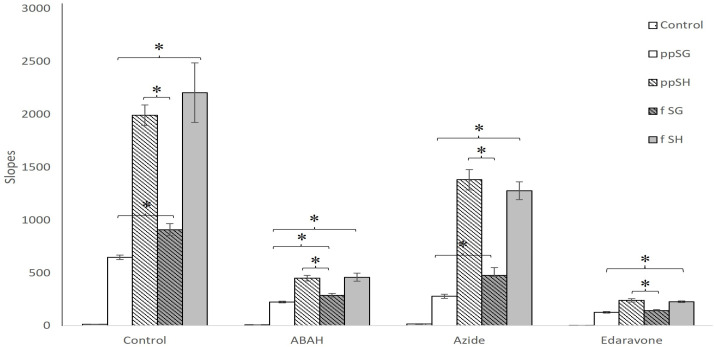
**Peroxidase-specific inhibitors reduce plasma-mediated oxidation.** The catalyzation of Amplex Red oxidation in the presence of general (azide) and peroxidase-specific substrates (ABAH, edaravone) were analyzed by HPLC using post-prandial sleeve gastrectomy (ppSG), post-prandial sham (ppSH), fasting sleeve (f SG) or fasting sham (f SH) plasma. Data presented as the mean ± SD, plasma pooled per sub-group and run in quadruplicate. Statistical significance determined at *p* < 0.05 (*).

We further tested whether the low H_2_O_2_ consumption rate observed for post-prandial SG plasma using the CBA assay was due to decreased peroxidatic activity. A redox probe, Amplex Red, was used to evaluate the peroxidatic activity of the plasma samples. As shown in Figure 4B, gavaged SG plasma showed the lowest ability to catalyze H_2_O_2_-induced Amplex Red oxidation, indicating the lowest peroxidatic activity of all the plasma samples tested. To further test the involvement of plasma peroxidase activity in the Amplex Red oxidation results, we subjected all the plasma samples to inhibitors of peroxidase activity: azide anion, ABAH, and edaravone. As illustrated in Figure 5, the specific peroxidase inhibitor, ABAH, and peroxidase substrate, edaravone, almost completely inhibited plasma-mediated Amplex Red oxidation (*p* < 0.01). The lower potency of peroxidase inhibitor, azide anion, may be due to the potential formation of azidyl radical, capable of oxidizing Amplex Red [20]. Taken together, the data obtained with the three redox probes suggest that the protective effects of post-prandial SG plasma against oxidative/metabolic stress may be due to a reduction in peroxidatic activity in the circulation, leading to lower cellular damage.

### 3.6. Products of HE Oxidation by Plasma Suggest MPO Involvement

**Figure 6:**
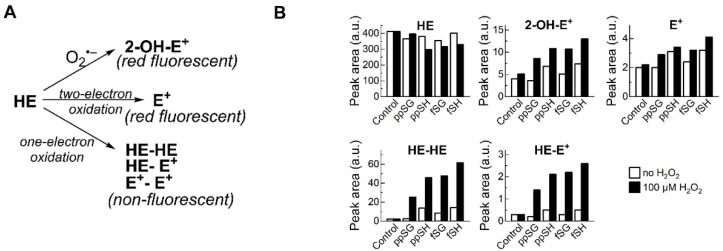
**Profiling hydroethidine oxidation products shows different peroxidatic activity of the plasma samples tested.** Effect of plasma samples on the extent of hydroethidine (HE, 40 µM) oxidation and the products formed in the absence and presence of H_2_O_2_ (100 µM) was tested utilizing plate reader- and HPLC-based measurements. (**A**) Scheme of HE oxidation. 2-hydroxyethidium (2-OH-E+) is a specific product for superoxide. Ethidium (E+) is a non-specific two-electron oxidation product. Dimeric products (HE-HE, HE-E+, E+-E+) are specific for one-electron oxidation. (**B**) Results of HPLC-based analyses of the incubation mixtures in the absence (control) and presence of different plasma samples (0.1% each), with and without H_2_O_2_. Determined HPLC peak areas for the HE probe and its different oxidation products.

In an attempt to further identify the H_2_O_2_-iduced oxidizing species formed, we performed HPLC analysis of the products produced from hydroethidine (HE) oxidation by H_2_O_2_, catalyzed by the plasma components from the sub-groups of Cohort 2. HE does not react directly with H_2_O_2_, but is susceptible to peroxidatic oxidation, with the formation of specific, dimeric products, detectable by HPLC [21]. Figure 6 shows the levels of HE and four oxidation products produced by the various plasma sub-groups over time. The pattern of products is highly suggestive of those produced from HE by MPO or other peroxidases, indicating that this might be the primary mechanism responsible for the reduced oxidative activity of post-prandial SG plasma [22].

### 3.7. Myeloperoxidase Protein Levels Are Decreased in Post-Prandial SG Plasma

**Figure 7:**
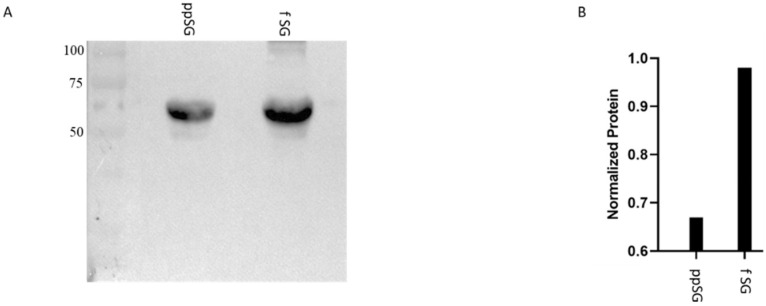
**Myeloperoxidase (MPO) protein content is decreased in post-prandial sleeve gastrectomy (ppSG) plasma.** (**A**) Western blot analysis of total MPO protein content in ppSG and fasting SG (f SG) plasma. (**B**) Density quantification of western bands.

Given the possibility of MPO involvement in oxidative protection in vitro, we examined the plasma MPO protein levels by Western blot from the post-prandial SG and fasting SG plasma (pooled plasma by sub-group). As shown in Figure 7A, the post-prandial SG plasma exhibited the lower levels of MPO. Densitometric analysis of the bands (Figure 7B) indicated that the MPO total protein in the post-prandial SG plasma was reduced by 35% compared to the fasting SG (Figure 7B). This result was consistent with the decreased peroxidatic activity observed (Figure 5 and Figure 6) and supports the hypothesis that the protective effects of post-prandial SG plasma against the oxidative insult are due to the nutrient-dependent reduction of circulating MPO after gastrectomy.

### 3.8. Plasma from MPO Knockout Rats Is Similarly Protective against In Vitro Oxidative/Metabolic Stress

**Figure 8:**
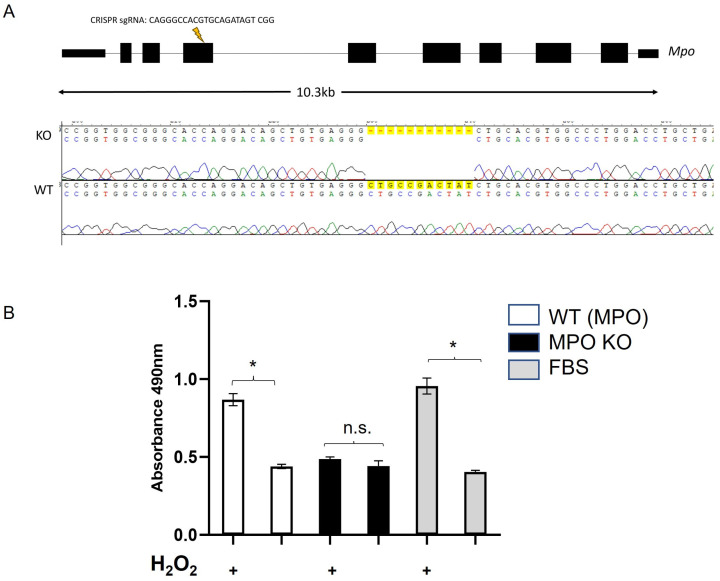
**Plasma from a myeloperoxidase (MPO) knockout (KO) rat is also protective against oxidative stress.** (**A**) Construction of a novel strain of Sprague Dawley rat was created through the injection of a SpCas9/sgRNA CRISPR ribonucleoprotein (RNP) targeting AGGGCCACGTGCAGATAGTCGG within the MPO gene of Crl:SD (Charles River Laboratories) rat embryos via pronuclear injection. One founder harboring an 11 base pair deletion (rn7: chr10:72595923-72595933) within exon 4 was back crossed with the parental strain to establish a stable genetic line. This strain is registered as SD-Mpoem1Mcwi (RGDID: 401717572). (**B**) Lactate dehydrogenase (LDH) secretion was measured from H9c2 cells pre-treated with 1% plasma in DMEM from MPO KO (n = 4) or wild type rats (n = 2). LDH release was measured as 490 nm absorbance under stressed (200 μM H_2_O_2_ and 10mM deoxy-glucose in DMEM) or unstressed conditions (high glucose DMEM). (*) represents a significant difference in LDH secretion from stressed compared to unstressed within each group (*p* < 0.05).

If decreased MPO activity is the primary mechanism for the oxidative protection effect of post-prandial SG plasma, then plasma from MPO knockout animals should behave similarly in the cell culture model of oxidative/metabolic stress. Figure 8A shows a cartoon and genetic sequencing of the construction of the MPO KO rat using CRISPR technology. As shown in Figure 8B, plasma from MPO knockout rats (n = 4), unlike wild type rats (n = 2), were resistant to cellular damage as assessed by LDH levels. Percent cytotoxicity is shown in Supplemental Figure S5. These data illustrate that the knockout of circulating MPO protein mimics the effect of post-prandial SG plasma in the prevention of oxidative/metabolic stress.

### 3.9. Plasma from MPO KO Rats Has a Rate of Hydrogen Peroxide Oxidation Similar to ppSG Plasma

**Figure 9:**
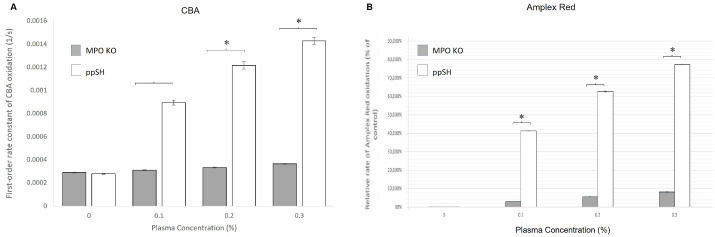
**Plasma from MPO KO rats has a rate of hydrogen peroxide oxidation similar to ppSG plasma.** (**A**) Oxidation rates of H_2_O_2_ by plasma ranging from 0 to 0.30% using coumarin-7-boronic acid (CBA). MPO KO rat plasma had the slowest rate of hydrogen peroxide oxidation at all plasma levels tested compared to post-prandial sham (ppSH). (*) represents a significant difference (*p* < 0.05). (**B**) Catalyzation of H_2_O_2_-induced Amplex Red oxidation using plasma ranging from 0 to 0.30% as detected by Amplex Red. As with the CBA probe, MPO KO plasma produces the slowest rate among plasma tested, indicating lower damaging oxygen free-radical production. Data presented as the mean ± SD, plasma pooled per sub-group and run in quadruplicate. (*) represents a significant difference (*p* < 0.05).

To determine whether plasma from an MPO KO rat would behave like that of post-prandial SG plasma in terms of consumption of H_2_O_2_, we tested the effect of plasma on the rate of H_2_O_2_-induced oxidation of CBA, using plasma doses from 0 to 0.30%. Based on a simple competition kinetics reasoning, we expected that ppSH consumption of H_2_O_2_ by plasma would result in faster completion of CBA oxidation compared to MPO KO plasma, reflected in an increased apparent first-order rate constant. The MPO KO plasma resulted in minimal CBA oxidation compared to the ppSH plasma (Figure 9A). This indicated that MPO KO plasma minimally consumed H_2_O_2_, and that MPO reduction may be a key component of the mechanism of oxidative protection as seen with the post-prandial SG plasma in Figure 4A. In a similar experiment, Figure 9B shows that the ability to catalyze H_2_O_2_-induced Amplex Red oxidation was also severely depressed, supporting the CBA data and the role of MPO in our surgical plasma studies.

## 4. Discussion

Bariatric surgery, including SG, has been shown to improve diastolic function independent of weight loss [13]. Our study focused on whether a circulating nutrient-stimulated factor from the gastrointestinal tract which acts on myotubles, as a surrogate for cardiomyocytes, plays a role in this functional improvement. We utilized an in vitro culture system in which H9c2 cells were exposed to H_2_O_2_ and deprivation of glucose to create oxidative/metabolic stress and tested if plasma from Zucker rats undergoing SG or sham surgery could alter cellular damage from the stress event. We found that only the plasma from post-prandial SG animals uniquely protected the H9c2 cells from oxidative/metabolic stress, in a weight loss-independent manner. We further identified that the protective effect of the post-prandial SG plasma on the H9c2 cells was extracellular, and likely due to an acute reduction in circulating MPO, and/or other plasma components exhibiting peroxidatic activity, following nutrient stimulation after gastrectomy. Future studies will need to confirm this effect of gastrectomy on MPO in humans; however, this suggests a novel role of surgical gastrectomy in disease states with pathogenesis based in oxidative stress, like HFpEF, by lowering oxidative free radicals and cellular damage.

MPO, a member of the heme peroxidase family of enzymes, is secreted primarily by neutrophils and monocytes as a part of the innate immunity and defense against pathogens with production of hypochlorous acid [23]. Although complete lack of MPO can increase the risk of infection, excess MPO-derived oxidative products are associated with multiple inflammatory disease states including obesity, insulin resistance, atherosclerosis, and heart failure [24,25,26]. Indeed, MPO levels are elevated in heart failure patients and associated with worse cardiac function [27,28]. Mouse studies have previously shown that MPO can contribute to detrimental cardiac remodeling and fibrosis, and conversely that MPO deficiency can preserve cardiac function in myocardial infarction models [29,30,31,32]. These studies indicate that a reduction in circulating MPO may be a contributing factor in the ability of SG to improve diastolic function. 

In an activated neutrophil, MPO is released into the phagosome; however, some MPO is released extracellularly and transported by albumin and neutrophil microparticles [33]. Plasma MPO and calprotectin levels are significantly increased in severely obese subjects as compared to healthy controls, likely from increased circulating neutrophils rather than from adipose or muscle tissue neutrophils. A prior study found bariatric surgery decreased calprotectin levels but not plasma MPO levels; however, this study only examined fasting MPO levels post-operatively [34]. Our findings suggest that MPO reduction may still be an important mechanism for decreased oxidant production post-operatively after bariatric surgery but specific to the post-prandial state. We do not know the cause for the acute reduction of circulating MPO in post-prandial plasma after SG but question a potential role for gut-mediated enzyme degradation or clearance. 

Metabolic surgery has wide-reaching effects on multiple disease states potentially centered on the reduction of systemic inflammation and metabolic stress. While we studied a myocyte model and are interested in HFpEF, our findings may have important implications for how metabolic surgery impacts other obesity-associated disease states. MPO has been implicated in the pathogenesis of several types of cancer including breast and colon cancer due to oxidative biproducts which are mutagenic [33]. MPO may also play a role in neurologic diseases such as Parkinson’s and Alzheimer’s dementia [23]. Many studies have found increased plasma MPO in patients with Alzheimer’s disease with co-localized MPO in beta-amyloid plaques [35]. A recent study found gastric bypass improved cognitive function in a mouse model of Alzheimer’s disease [36]. Mechanistic studies are needed to understand how bariatric surgery improves cognitive function and if decreased oxidative stress and free radical damage are involved.

The strengths of this study are that we were able to validate the effect of SG plasma from Zucker rats from two separate surgical cohorts performed at different times and from different litters increasing reproducibility. Further, the use of a novel MPO KO rat serves as strong support of the role of MPO in our cellular model. The study limitations include the use of the Zucker rat as a single genetic mutation of obesity, limiting possible translational relevance to clinical SG and redox balance states post-operatively. In addition, H9c2 cells are primitive rat, muscle cells rather than mature cardiomyocytes. H9c2 cells were utilized due to the number of different studies required over time under different cellular conditions but limits direct application to the cardiomyocyte; therefore, future studies are needed both in primary rat cardiomyocytes and with human samples using isolated pluripotent stem cell-differentiated cardiomyocytes to verify oxidative/metabolic protection, and if possible with mature cardiomyocytes as well. Finally, due to the number of studies required, we also had to pool blood samples by group, rather than run samples as individual animals, increasing the risk of a type 1 statistical error. 

## 5. Conclusions 

This study has identified that a potential mechanism for the improvement in cardiac function after SG is reduction of circulating MPO, or other peroxidases, which lowers the oxidative/metabolic stress of myocytes and is dependent on nutrient-stimulation of the gastrointestinal tract. Future studies are needed to confirm this effect is present clinically and determine the mechanism for how a gastrectomy lowers MPO in the systemic circulation upon nutrient stimulation. If this surgical effect is confirmed in humans, SG may be a unique surgical treatment for multiple diseases with a pathogenesis of inflammation and oxidative damage, including obesity-associated HFpEF.

## Data Availability

Data are contained within the article and Supplementary Materials.

## Supplementary Materials

The following supporting information can be downloaded at: https://www.mdpi.com/article/10.3390/nu15224776/s1, Supplement Figure S1: LDH Secretion increases in response to oxidative stress. Supplement Figure S2: H_2_O_2_ dose curves in H9c2 cells. A high level of toxicity is observed above 200 μM. Supplement Figure S3: Plasma from Zucker rats undergoing sleeve gastrectomy (SG) is protective against oxidative/metabolic stress and weight loss-independent. Supplement Figure S4: Protection against oxidative/metabolic stress by sleeve gastrectomy (SG) plasma is acute and nutrient dependent. Supplement Figure S5: Plasma from a myeloperoxidase (MPO) knockout (KO) rat is also protective against oxidative stress.

## Abbreviations

ABAH	aminobenzoic acid hydrazide
CBA	coumarin-7-boronic acid
DMEM	Dulbecco’s Modified Eagle Medium
HFpEF	heart failure with preserved ejection fraction
HE	hydroethidine
H_2_O_2_	hydrogen peroxide
KO	knockout
LDH	lactate dehydrogenase
MPO	myeloperoxidase
PF	pair-fed
PP	post-prandial
SH	sham
SG	sleeve gastrectomy
