# Supplementary material for: Sleeve Gastrectomy Provides Cardioprotection from Oxidative Stress In Vitro Due to Reduction of Circulating Myeloperoxidase
Source: Nutrients. 2023 Nov 14;15(22):4776. doi: 10.3390/nu15224776 (PMC10675224; doi:10.3390/nu15224776)
Supplement: Supplementary file 1 [file nutrients-15-04776-s001.zip › nutrients-2649860-supplementary.pdf]

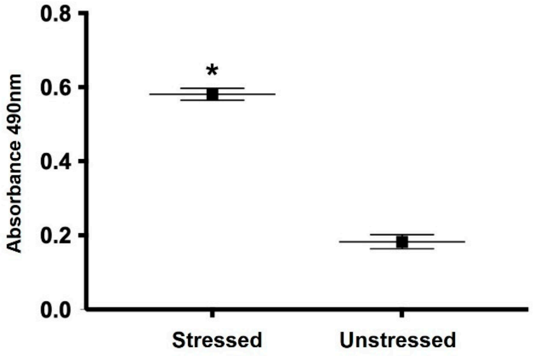

Figure S1. LDH Secretion increases in response to oxidative stress. 1 hour of stress exposure [1% FBS in DMEM (*no glucose*), deoxyglucose (10mM) and H_20_2 (100µM)] leads to a significant increase in LDH release in stressed H9c2 cells compared to unstressed controls. Raw data values are represented as the mean ± SD, n=3 (*p<0.01).

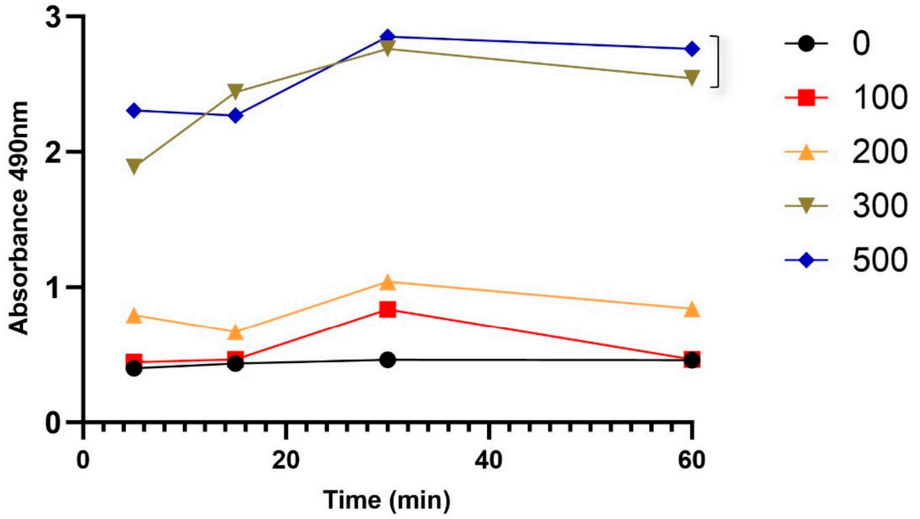

Figure S2. H_2O_2 dose curves in H9c2 cells. A high level of toxicity is observed above 200uM (bracket).

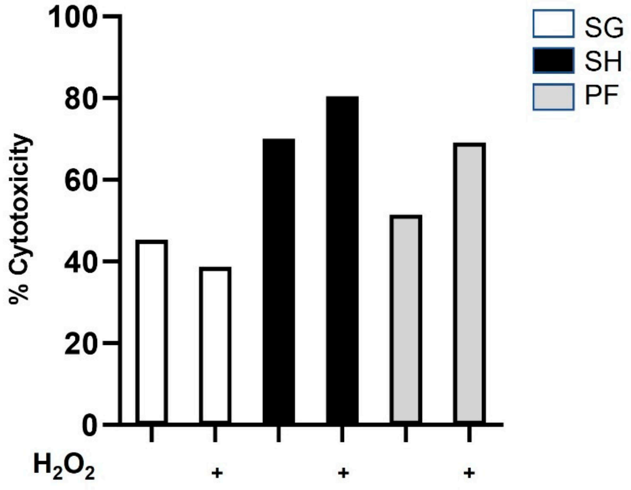

Figure S3. Plasma from Zucker rats undergoing sleeve gastrectomy (SG) is protective against oxidative/metabolic stress and weight-loss independent. Percent toxicity as measured by lactate dehydrogenase **(**LDH) secretion at 490 nm absorbance from H9c2 cells pre-treated with 1% plasma in DMEM from obese Zucker rats who had undergone SG (SG), ad-lib sham (SH), or pair-fed sham (PF) surgeries. LDH release was measured as 490 nm absorbance under stressed (200uM H_2O_2 and 10mM deoxy-glucose in DMEM) or unstressed conditions (high glucose DMEM).

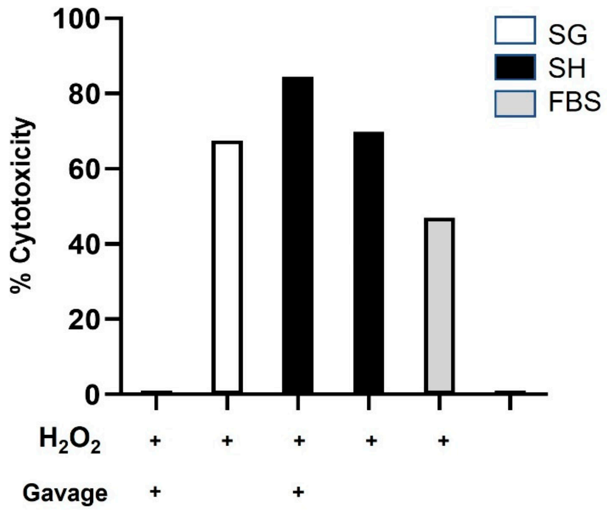

Figure S4. Protection against oxidative/metabolic stress by sleeve gastrectomy (SG) plasma is acute and nutrient dependent. Percent toxicity as measured by lactate dehydrogenase **(**LDH) secretion at 490 nm absorbance from H9c2 cells pre-treated with 1% plasma in DMEM from obese Zucker rats who had undergone SG (SG) or Sham (SH) surgery under fasting or 15 minutes following a 10% glucose gavage with FBS as a control. The sub-groups were tested under stress (200uM H_2O_2 and 10mM deoxy-glucose in DMEM) or unstressed conditions (high glucose DMEM).

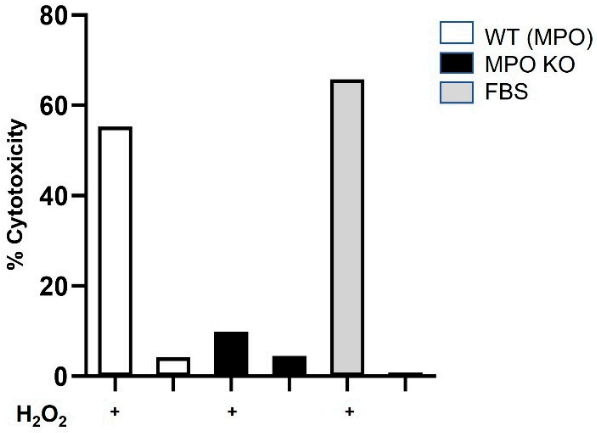

Figure S5. Plasma from a myeloperoxidase (MPO) knockout (KO) rat is also protective against oxidative stress. Lactate dehydrogenase (LDH) secretion was measured from H9c2 cells pre-treated with 1% plasma in DMEM from MPO KO or wild type rats. LDH release was measured as 490 nm absorbance under stressed (200uM H_2O_2 and 10mM deoxy-glucose in DMEM) or unstressed conditions (high glucose DMEM). (*) represents a significant difference in LDH secretion from stressed compared to unstressed within each group ($p<0.05$).
